# The Antwerp Vestibular Compensation Index (AVeCI): an index for vestibular compensation estimation, based on functional balance performance

**DOI:** 10.1007/s00405-020-06192-4

**Published:** 2020-08-05

**Authors:** Evi Verbecque, Floris L. Wuyts, Robby Vanspauwen, Vincent Van Rompaey, Paul Van de Heyning, Luc Vereeck

**Affiliations:** 1 Department of Rehabilitation Sciences and Physiotherapy/Movant, Faculty of Medicine and Health Sciences, University of Antwerp, Universiteitsplein 1, 2610 Wilrijk, Belgium; 2 Rehabilitation Research Centre (REVAL), Rehabilitation Sciences and Physiotherapy, Hasselt University, Agoralaan Building A, 3590 Diepenbeek, Belgium; 3 Lab for Equilibrium Investigations and Aerospace (LEIA), University of Antwerp, Universiteitsplein 1, 2610 Wilrijk, Belgium; 4 European Institute for Otorhinolaryngology, GZA Hospitals Antwerp, Wilrijk, Belgium; 5 Department of Otorhinolaryngology and Head and Neck Surgery, Antwerp University Hospital, Edegem, Belgium; 6 Department of Translational Neurosciences, Faculty of Medicine and Health Sciences, University of Antwerp, Universiteitsplein 1, Campus Drie Eiken, 2610 Wilrijk, Belgium; 7 Multidisciplinary Motor Centre Antwerp (M2OCEAN), University of Antwerp, Universiteitsplein 1, Campus Drie Eiken, D.R.311, 2610 Wilrijk, Belgium

**Keywords:** Postural balance, Vestibular function tests, Adult, Functional assessment

## Abstract

**Purpose:**

To create an index that is a measure of the amount of vestibular compensation and for which only functional balance performance is needed.

**Methods:**

The medical charts of 62 eligible peripheral vestibular dysfunction (PVD) patients were analyzed retrospectively. To be included, the following vestibulo-ocular reflex (VOR) and balance performance data had to be available: (1) caloric and sinusoidal harmonic acceleration test (SHA) and (2) standing balance sum-eyes closed (SBS-EC), Timed Up and Go Test and Dynamic Gait Index. Patients were divided into three groups: normal caloric- and SHA test (group 1), abnormal caloric- and normal SHA test (group 2, PVD compensated) and abnormal caloric- and SHA test (group 3, PVD uncompensated). Next to the use of non-parametric tests to study the VOR and balance variables, logistic regression was used to identify the balance measures that predict whether PVD patients were compensated or uncompensated. This resulted also in the construction of a continuous measure representing the degree of compensation.

**Results:**

Logistic regression identified SBS-EC and age to classify uncompensated from compensated patients with sensitivity of 83.9% and specificity of 72.4%. Then an index was created, called the Antwerp Vestibular Compensation Index, AVeCI = − 50 + age × 0.486 + SBS-EC × 0.421. A patient belongs to the uncompensated group when AVeCI < 0 and to the compensated group when AVeCI > 0, with respective group means of − 5 and 5.

**Conclusion:**

AVeCI stages the degree of compensation of PVD patients and can serve to evaluate rehabilitation effects.

## Introduction

Patients with acute peripheral vestibular dysfunction (PVD) complain about vertigo, nausea, spatial disorientation, gaze- and postural instability in the first few days after the onset of vestibular function loss [1–5]. These symptoms result from deficient vestibulo-ocular reflex (VOR) and vestibulo-spinal reflex (VSR) systems and abnormally activated vestibulo-thalamo-cortical pathways [1, 2, 4]. In the majority of patients, these symptoms tend to ameliorate with time as a result of central vestibular compensation, i.e. the central nervous system adapts to the changed vestibular input and/or substitutes for it with other more reliable information sources [3, 5]. During this period, patients no longer experience symptoms at rest, but they still complain of head-movement-induced gaze- and postural instability [3, 5].

If central vestibular compensation occurs adequately, whether it emerges naturally or is facilitated by therapeutic interventions such as vestibular rehabilitation (VR) [6], peripheral vestibular dysfunctions may remain detectable (e.g. abnormal caloric test [7] or vestibular evoked myogenic potentials test (VEMP) [8]), while the VOR function appears to be recovered. To map VOR recovery, the traditional SHA test can be used [7] to map low to middle frequency responses, whereas the caloric test maps very low frequencies and the video Head Impulse Test (vHIT) represents high frequency responses [9, 10]. The VOR performance is typically characterized by gain, asymmetry and phase [7]. Gain is the ratio of the compensating eye movement velocity compared to the velocity of the head movement, while VOR asymmetry is based on the comparison between ipsi-lesioned eye velocity and contra-lesioned eye velocity. The VOR phase is determined by the time difference between head velocity and eye velocity [1, 7]. Acute PVD caused by vestibular neuritis for example, results in a decreased VOR gain, increased asymmetry and increased phase. Recovery of the VOR function, i.e. adequate compensation is therefore reflected by an increased gain, decreased asymmetry and reduction of the phase, although the latter is more difficult to calculate when permanent peripheral function loss is present. When adequate compensation occurs, the delay within the vestibular system decreases in such way that the typical visible catch-up saccades during head impulses become covert [11].

Although static symptoms such as spontaneous nystagmus and postural misalignment improve and eventually disappear spontaneously, more dynamic symptoms such as motion sensitivity, balance deficits and spatial disorientation can remain for several weeks to months [12]. Unfortunately, up to 50% of the patients report remaining symptoms to some degree after one year [12, 13]. In the chronic stage, some patients with unilateral PVD have symptoms that resemble those from patients with bilateral PVD [11]. In about 20% of the chronic unilateral PVD patients, postural instability tends to persist and vertical oscillopsia occurs, highlighting the remaining presence of both VSR and VOR deficits [11]. These symptoms and complaints highly incapacitate the patients’ daily functioning, for which they can receive VR. Although VR has proven to be effective in patients with PVD [14], clear indications for its application are still lacking, which can be ascribed to the conflicting results with respect to the vestibulometric outcome parameters [12].

Though the functional relationship between VSR and VOR is recognized, evidence for this assumption is lacking due to conflicting results [1–3, 15]. So far, weak [1–3, 15] to moderate [3, 15] correlations have been reported between balance performance and caloric labyrinth asymmetry [3, 15] or VOR gain [1, 2, 15] in patients with PVD. The caloric test (low frequency), the SHA test (low to middle frequency) and the vHIT (high frequency) address different aspects of VOR function which may explain the varying results. Similarly, to evaluate the VSR, a large variety of test methods is available, each emphasizing other task constraints of balance control and/or pointing at other factors influencing performance. For example, the type of task influences which balance mechanism is mainly needed to remain stable. Some tests involve sensory perturbations whereas other tasks investigate the effect of an increased cognitive load [16].

Another aspect illustrating the vague relationship between VOR and VSR function is the considerable disagreement with respect to the value of balance tests being able to discriminate between healthy individuals and patients with impaired VOR-function. For example, the sensitivity and specificity to indicate VOR dysfunction was respectively 61.3% and 58.3% for the Romberg test eyes open and eyes closed [17], 75% and 77% for the Timed Up and Go Test (TUG), 75% and 75% for the Berg Balance Scale, 78% and 75% for the Dynamic Gait Index (DGI) and 85% and 77% for condition 5 of the sensory organization test (SOT-5) [18]. Cohen and Kimball [18] found that combining the SOT-5 with a functional measure such as the TUG or DGI allowed the diagnostic accuracy of the applied tests to increase, i.e. a 90% sensitivity and 67% specificity was reached. The lacking accuracy of the balance tests to identify uncompensated PVD patients may be due to the heterogeneity of the procedures to assess the vestibular function in the populations studied. Where Jacobson et al. [17] included PVD patients with a positive or negative result on either the caloric test or the cervical VEMP, Cohen and Kimball [18] included patients with PVD if they had at least one positive result on either the caloric test, the SHA test or the Dix–Hallpike maneuver. This means that the investigated samples may consist of different types of vestibular pathologies, but also that both uncompensated and well-compensated patients were part of the same group, potentially distorting the results.

While VOR gain, asymmetry and phase are appropriate identifiers of VOR compensation, these variables are standard not readily available in most clinical and rehabilitation settings throughout the world. More accessible though are functional balance performance tests since these require no expensive equipment.

The aim of this study is to investigate whether PVD patients could be allocated appropriately as compensated or not, solely relying on functional balance performance. Classification by itself is not the aim but we aimed for a tool that allows therapists to measure the effect of vestibular rehabilitation and adjust their therapy if necessary. A classification method such as logistic regression can yield a tool to stage the degree of being part to one or the other group on a linear scale. This method proved to be very successful to identify hoarseness in patient with voice problems. The Dysphonia Severity Index, published in 2000, has become one of the standard tools worldwide today to evaluate a patient’s voice, and assess the given therapy [19].

## Methods

### Participants and study design

In this retrospective study, approved by the local ethical committee, anonymized patient charts were reviewed and analyzed. Patients referred to a tertiary otorhinolaryngology department at the local university hospital between 2001 and 2007 for an additional physiotherapeutic assessment because of complaints of instability or dizziness. To be included in the analyses, data on both vestibular function and functional balance testing had to be available. From the available patient files (*n* = 175), results from 62 patients were eligible for analyses [mean age (SD) 52.3 (11.9) years; 35 males]. Reason for exclusion were: incomplete ENG data (*n* = 60), balance testing more than two weeks after ENG assessment (*n* = 46) and a central lesion (*n* = 7).

### Procedures

#### Caloric testing

The external ear canals were consecutively irrigated with 180 ml of warm (44 °C) and cold (30 °C) water for 30 s with open loop irrigation (warm right, warm left, cold right, cold left). In a nearly complete darkened room patients closed the eyes during testing and performed mental tasks. To allow temperature stabilization in the labyrinth, a pause of 5 min was introduced between irrigations of the same ear. For both binaural bithermal caloric testing and the SHA test computerized electronystagmography was used to register eye movements. A detailed description on the applied methodology has been described elsewhere [7].

Based on the slow component velocity (SCV, °/s), obtained during the maximal response of a caloric irrigation, Jongkees’ formula was used to calculate the percentage of labyrinth asymmetries. This labyrinth asymmetry is considered normal if the difference between both ears was less than 19% [7], based on a normative study performed in the same clinical setting, with exactly the same ENG procedure. Caloric testing has shown to be a valid measure to assess labyrinth asymmetries [20].

#### Sinusoidal Harmonic Acceleration test (SHA)

Subjects were seated in a servo-controlled motorized chair that was rotated sinusoidally around an earth vertical axis [7]. The chair was accelerated to a peak velocity of 50°/s and the frequency of the sinusoidal acceleration was 0.05 Hz. The test duration was 2 min. An angular rate sensor, attached to the subject’s head, recorded the head movements. The test was performed in total darkness with the eyes closed and patients were requested to perform mental tasks for mental alertness. Based on the slow phase velocity component, VOR gain, -asymmetry and -phase were calculated. The VOR gain is a measure of VOR performance calculated by the velocity of the correcting eye movement divided by the velocity of the head (normal values between 0.29 and 0.87) [7]. The VOR asymmetry stands for the percentage difference between the peak slow component eye velocities of the nystagmi to the left and right (normal value < 22%) [7]. The phase comprises the angle of the response which is a representation of the time difference between the eye and the head velocity and therefore a measure of the delay in the vestibular system (normal values between − 1° and 18°) [7]. The SHA is a valid VOR test that is clinically useful for the observation of a patient’ s progress through central compensation after unilateral dysfunction [20–22].

Based on the results on the caloric test and the SHA, three groups were composed. Group 1 consisted of patients that presented with a normal ENG, i.e. caloric symmetry and VOR gain, -asymmetry, and phase values within normal limits. If patients exhibited caloric asymmetry and normal VOR gain, asymmetry and -phase, they were classified in group 2 (peripheral loss, compensated). If patients showed caloric asymmetry and an abnormal result in either VOR gain, -asymmetry or -phase, they were assigned to group 3 (peripheral loss, uncompensated). For the logistic regression, groups 1 and 2 were merged.

#### Dizziness Handicap Inventory

The Dizziness Handicap Inventory (DHI) was used to assess the degree to which vestibular dysfunction subjectively affects overall activities of daily life [23, 24]. The questionnaire comprises 25 questions that the patient must answer with “always” (4 points), “sometimes” (2 points) or “no” (0 points) [23, 24]. The ordinal item scores were added up resulting in a maximum of 100, with 0–30 points indicating a mild handicap as a consequence of dizziness and instability, 31–60 points moderate handicap and 61–100 severe handicap [23]. The DHI is the most commonly used patient-reported outcome measure in clinical vestibular research [25]. A validated Flemish version of the DHI was used [26–28].

#### Standing balance

In clinical practice many stand-alone clinical tests such as classic Romberg with Jendrassik maneuver (RJ), standing on foam (SOF), tandem stance (TS) and single les stance (SLS) with eyes open (EO) and/or eyes closed (EC) are currently used to assess standing balance in vestibular patients. As the effect of age on performance, when using one single balance test, might interfere with vestibular pathology we investigated both the single tests and a combination of these tests [29]. A standardized foam pad with medium density (60 kg/cm^3^) was used (45 × 45 × 12 cm, NeuroCom International Inc. Clackamas, USA).

Patients were instructed to stand for 30s in each of the following seven conditions: RJ EC, SOF EO, SOF EC, TS EO, TS EC, SLS EO, SLS EC [29]. A digital stopwatch was used for time measurements. The best of three trials was considered for analysis.

For each single balance test scores (seconds) varied between 0 and 30 with higher scores indicating better balance. The scores of the single balance tests in the EC conditions were summed, resulting in the standing balance sum eyes closed (SBS-EC). This variable was selected for logistic regression, because it was more sensitive for vestibular disorders. Hence, the SBS-EC scores ranged between 0 and 120 s, with higher scores indicating better standing balance [29].

#### The Timed Up and Go Test (TUG)

The TUG was administered following the protocol by Podsiadlo and Richardson [30] using a standard chair with arm- and back rests and seat height of 46 cm. Subjects performed the TUG as fast as possible but safely. Timing was started at the cue “go” and stopped when the patient sat down again on the chair with their back against the back rest after walking for 3 m and turning back. A digital stopwatch was used for time measurements. All patients performed the TUG three times when turning in the preferred direction and again three times when turning in the opposite direction [29]. The best time was used as the final result and was considered normal if it was less than 10s [30]. Slower scores on the TUG (> 11.1 s) correlated with reports of falls in persons with vestibular dysfunction [31]. The TUG has moderate sensitivity and specificity in identifying individuals with disequilibrium due to vestibular impairments [18, 31].

#### The Dynamic Gait Index (DGI)

During DGI assessment, the patient performs eight walking conditions [32]: walking on a level surface, walking with changing walking speed, walking with horizontal and vertical head turns, making a 180° turn and stop after walking, stepping over and around objects and stair climbing. Each condition is rated with a 4-point rating scale (minimum 0 and maximum 3 points), in which three points represents best performance, resulting in a total score of maximum 24 points [32]. A score of less than 19 points indicates risk of falling [31, 33]. Lower scores on the DGI correlated with reports of falls in persons with vestibular dysfunction [31]. The DGI’s sensitivity and specificity is moderate to identify individuals with disequilibrium due to vestibular impairments [18, 31].

### Data analysis and statistical analysis

Statistical analyses were performed with SPSS 25.0 for windows. Normal distribution was verified using the Kolmogorov–Smirnov test. The sample was described with minima, maxima, medians and interquartile ranges (IQR) of age, body mass index (BMI), the caloric SCV asymmetry (%), SHA VOR gain, SHA VOR asymmetry (%) and SHA VOR phase (°), DHI (points), RJ EC (s), SOF EO (s), SOF EC (s), TS EO (s), TS EC (s), SLS EO (s), SLS EC (s), SBS-EC (s), TUG (s), DGI (points) and the distribution of sex, side of the lesion and the etiology.

To determine how balance performance relates to VOR recovery in patients with PVD, Spearman’s rho correlations (*ρ*) were calculated between variables. Correlation coefficients were interpreted as: very high (0.9–1.00), high (0.7–0.9), moderate (0.5–0.7), low (0.3–0.5) or negligible (< 0.3) [34].

Investigations on the most suited balance test to identify uncompensated PVD patients were performed in two steps. First, differences in balance performance between the three groups were investigated with the Kruskal–Wallis test, followed by pairwise comparison with the Mann Whitney *U* test using Bonferroni correction for multiple comparisons. Level of significance was set at *α *= 0.05.

If present, the differences in vestibular and balance function between the groups indicated group 3 (PVD uncompensated) was distinguishable from group 1 (normal) and/or group 2 (PVD compensated). Therefore group 1 and 2 were considered one group (group 1 and 2) in further analyses. Subsequently, stepwise backward logistic regression analysis was performed to determine the optimal combination of balance measures that separates uncompensated PVD patients from those who have normal vestibular function or are compensated. All balance measures were used as input variables. The outcome resulting in the highest sensitivity and specificity was selected. A standard logistic regression procedure results in a function *f* = *c*_0_ +* c*_1_ × *X*_1_ + *c*_2_ × *X*_2_ + ⋯ + *c*_*n*_ × *X*_*n*_. In this function, the coefficients *c*_1_, *c*_2_ etc. are determined such that the combination with the variables *X*_1_, *X*_2_ etc. yield the best classification matrix with the highest sensitivity and specificity. When the logistic regression is performed stepwise, only those variables *X*_1_, *X*_2_ that are contributing to the best classification are selected. Hence, the selection in our study of SBS-EC and age, although many other as well as more variables could have been retained. When the logistic regression yields for example *f* = − 6.9 + 0.07 × age + 0.06 × SBS-EC, this means that for a given patient who is 60 years old and has a SBS-EC of 120 s (the best performance), the function *f* = −6.9 + 0.07 × 60 + 0.06 × 120 = 4.5. Then, the logistic regression transforms this value into *F* = 1/(1 + exp(− *f*)), and this yields then *f* = 0.99. Whereas *f* can vary between − infinity to + infinity, the function *f* varies between 0 and 1. If for a given patient *f* > 0, this yields that *f* > 0.5, and this patient is attributed to the compensated group. If however *f* < 0, and hence *f* < 0.5, this patient is classified to the uncompensated group. This model is compared with the actual status of the patient, and based on iterations, the best combination of variables and coefficients is determined. Optimally, a 100% sensitivity and specificity would be ideal, but this is seldom the reality. Rather than focusing on classification, we introduce an index which is based on the function *f*. We calculated the average *f* for each group and rescaled that to − 5 and + 5 for the both uncompensated and compensated group. This yields the final equation.

## Results

As shown in Table 1, all balance measures, except for the RJ EC, had low to high correlations with age. The caloric SCV asymmetry had low correlations with SOF EC, TS EC, SLS EC and SBS-EC (*ρ* = − 0.379/− 0.306/− 0.401/− 0.339). The SHA gain had a significant but negligible correlation with the DGI (*ρ* = 0.296). The SHA gain asymmetry did not correlate with any of the balance measures. The SHA phase had a low correlation with the RJ EC, SOF EC, TS EC and SLS EC (*ρ* = − 0.282/− 0.462/− 0.431/− 0.400), but a moderate correlation with the SBS-EC (*ρ* = − 0.531).

**Table 1:** Spearman’s *ρ* correlation coefficients between balance measures and vestibular function measures for all patients

	Age		Caloric asymmetry (%)		SHA gain		SHA gain asymmetry (%)		SHA phase (°)	
	*ρ*	*p* value	*ρ*	*p* value	*ρ*	*p* value	*ρ*	*p* value	*ρ*	*p* value
Age			0.155	0.229	− 0.146	0.259	− 0.138	0.285	0.165	0.204
Romberg Jendrassik EC (s)	− 0.211	0.100	− 0.059	0.651	0.204	0.111	0.148	0.251	**− 0.282**	**0.028**
Standing on foam EO (s)	**− 0.362**	**0.004**	− 0.111	0.391	0.167	0.196	0.097	0.452	− 0.072	0.580
Standing on foam EC (s)	**− 0.665**	**< 0.001**	**− 0.397**	**0.001**	− 0.040	0.760	0.195	0.130	**− 0.462**	**< 0.001**
Tandem stance EO (s)	**− 0.385**	**0.002**	− 0.144	0.274	0.102	0.439	0.156	0.234	− 0.365	0.005
Tandem stance EC (s)	**− 0.743**	**< 0.001**	**− 0.306**	**0.018**	0.038	0.776	0.142	0.278	**− 0.431**	**0.001**
Single leg stance EO (s)	**− 0.702**	**< 0.001**	− 0.114	0.379	0.174	0.177	0.142	0.271	− 0.159	0.221
Single leg stance EC (s)	**− 0.704**	**< 0.001**	**− 0.401**	**0.001**	− 0.002	0.987	0.171	0.183	**− 0.400**	**0.001**
Standing balance sum EC (s)	**− 0.737**	**< 0.001**	**− 0.379**	**0.003**	0.225	0.085	− 0.045	0.734	**− 0.531**	**< 0.001**
TUG (s)	**0.482**	**< 0.001**	0.026	0.839	− 0.154	0.233	− 0.089	0.491	− 0.005	0.967
DGI (points)	**− 0.398**	**0.001**	− 0.186	0.148	**0.269**	**0.034**	0.023	0.862	− 0.186	0.151

Significant values are shown in bold

*SHA* sinusoidal harmonic acceleration test. *EO* eyes open, *EC* eyes closed, *TUG* Timed Up and Go Test, *DGI* Dynamic Gait Index

Table 2 depicts the patient characteristics of the three groups separately. As defined by the group composition, the median values of the caloric SCV asymmetry, the SHA VOR gain, the SHA VOR phase differ significantly between groups. Compared to group 1, the caloric SCV asymmetry was significantly larger for group 2 (group 1–2, *p* = 0.002) and group 3 (group 1–3, *p* < 0.001). Group 3 had significantly lower SHA gain values compared to the other groups (group 1–3, *p* = 0.036; group 2–3, *p* = 0.014) and larger SHA phase values (group 1–3, *p* < 0.001; group 2–3, *p *< 0.001). Kruskal–Wallis indicated significant differences for SOF EC, SLS EC and SBS-EC between the three groups. Post-hoc analysis showed that patients in group 3 had shorter SOF EC time compared to group 1 (*p* = 0.020) and group 2 (*p* = 0.007), but also that their SBS-EC was significantly lower than that of group 2 (*p* = 0.006) and group 1 (*p* = 0.019).

**Table 2:** Description of the sample as a function of group assignment based on type of vestibular loss

	Group 1: normal vestibular function					Group 2: peripheral loss, compensated					Group 3: peripheral loss, uncompensated^b^					Differences between groups	
	*N*	Median	IQR	Min	Max	*N*	Median	IQR	Min	Max	*N*	Median	IQR	Min	Max		*p* value
Age	9	52.5	31.9	28.7	79.6	20	50.8	15.5	29.7	75.6	33	55.8	12.6	34.7	72.5		0.270
BMI	9	21.2	6.8	17.0	28.7	17	24.9	6.7	19.4	37.7	33	24.2	4.0	17.6	32.0		0.255
Dizziness Handicap Inventory	9	26.0	32.0	4.0	80.0	20	14.0	20.0	0.0	68.0	33	24.0	50.0	0.0	80.0	2 < 3*	**0.019**
Male	4					14					17						
Affected side																	
Unilateral	6					20					31						
Bilateral	1										2						
Unknown	2																
Etiology																	
Vestibular schwannoma^a^	5					17					27						
Selective vestibular neurectomy											1						
Menière’s disease	1										1						
Meningioma						1											
Post-traumatic						1											
Multiple sensory deficit	1					1											
Unknown																	
Chronic dizziness											2						
Chronic instability	2										2						
Caloric test—asymmetry (%)	9	9.0	13.0	1.0	18.0	20	40.0	34.0	20.0	90.0	33	67.0	28.0	20.0	100.0	1 < 2, 3*	**< 0.001**
SHA—asymmetry (%)	9	5.0	10.0	0.0	18.0	20	11.0	13.5	0.0	20.0	33	12.0	14.5	0.0	36.0		0.142
SHA—gain	9	0.60	0.41	0.32	0.96	20	0.60	0.38	0.31	1.06	33	0.33	0.35	0.04	0.94	3 < 1, 2*	**0.004**
SHA—phase (°)	9	8.0	12.0	1.0	18.0	20	13.5	4.8	1.0	17.0	32	21.5	9.8	2.0	61.0	3 > 1, 2*	**< 0.001**
Romberg Jendrassik EC (s)	9	30.00	0	30.00	30.00	20	30.00	0	30.00	30.00	31	30.00	0	1.27	30.00		0.096
Standing on foam EO (s)	9	30.00	0	30.00	30.00	20	30.00	0	13.99	30.00	31	30.00	0	30.00	30.00		0.571
Standing on foam EC (s)	9	30.00	24.66	4.34	30.00	20	30.00	23.79	0	30.00	31	4.10	11.09	0	30.00	3 < 1, 2*	**0.002**
Tandem stance EO (s)	9	30.00	0	13.70	30.00	20	30.00	0	8.15	30.00	31	30.00	0	6.71	30.00		0.095
Tandem stance EC (s)	9	6.30	21.49	2.13	30.00	20	6.41	22.42	0	30.00	31	3.72	6.25	0	30.00		0.085
Single leg stance EO (s)	9	30.00	9.70	3.00	30.00	20	30.00	18.64	11.36	30.00	31	30.00	18.41	0	30.00		0.195
Single leg stance EC (s)	9	6.77	28.10	0	30.00	20	4.31	9.59	0	30.00	31	2.00	4.00	0	12.70		**0.014**
Standing balance sum-EC (s)	9	75.39	74.03	36.78	120.00	20	70.05	55.04	30.00	120.00	31	39.09	20.91	2.98	102.70	3 < 1, 2*	**0.001**
Timed Up and Go Test (s)	9	9.08	2.30	6.56	10.34	20	7.75	1.69	6.34	10.87	33	8.38	2.21	6.13	23.10		0.699
Dynamic Gait Index (points /24)	9	23.00	2.0	16.0	24.0	20	23.00	2.0	16.0	24.0	33	22.00	5.0	3.0	24.0		0.109

Bold values are statistically significant (Kruskal Wallis; *p* < 0.05)

*N* number of patients, *IQR* interquartile range, *Min* minimum, *Max* maximum, *EO* eyes open, *EC* eyes closed, *SHA* sinusoidal harmonic acceleration

*Significant difference between groups after Bonferroni correction

^a^90% of these patients was evaluated preoperatively

^b^6/33 patients had deviant SHA VOR asymmetry, 13/33 patients had deviant SHA VOR gain, 27/33 patients had deviant SHA VOR phase

Logistic regression analysis defined SBS-EC and age as indicators for uncompensated PVD with an 83.9% sensitivity and a 72.4% specificity (*f* = −6.912 + 0.067 × age + 0.058 × SBS-EC). Based on this the index AVeCI was calculated as AVeCI = − 50 + 0.486 × age + 0.421 × SBS-EC.

## Discussion

The initial aim of the present study was to examine the relationship between selected VOR and VSR variables and to the investigate whether functional balance performance can be used to identify uncompensated PVD patients. Standing balance with EC, whether assessed on foam, feet positioned in tandem or on one leg, was related to labyrinthine asymmetry. This indicates that decreased unilateral low frequency excitability of the labyrinth induces lateropulsion resulting in poor standing balance in darkness. The low correlations between the caloric SCV asymmetry and standing balance are in line with the literature [3, 15]. Similarly, there were low to moderate correlations between these tests, SBS-EC included, and the SHA VOR phase (Table 1). The majority of the uncompensated PVD patients had a deviant SHA VOR phase (*n* = 27/33). This provides an indication for the differences we found in the standing balance performances across groups, distinguishing in particular the uncompensated PVD patients from the others (normal vestibular function and compensated PVD). These identified differences between the three groups are not in line with previous findings. Gouveris et al. [35] also investigated balance performance, using condition 5 (eyes closed on moving platform) and condition 6 (moving visual surround and moving platform) of the SOT, in three similarly composed groups. They found that patients with normal vestibular function on both caloric (labyrinthine asymmetry < 25%) and SHA (directional preponderance ≤ 20%) could be distinguished from those with confirmed caloric PVD with(out) central compensation. The authors therefore argued that these SOT conditions could be used to evaluate the peripheral integrity of the horizontal semi-circular canal.

The different findings in our study are presumably the result of the group composition, i.e. the VOR phase involvement, which was not reported by Gouveris et al. [35]. Based on the current results, it seems that an uncompensated system works inefficiently and requires more time to generate an appropriate response. In order to compensate for the loss of vestibular information, supplementary (visual, somatosensory, auditory) information and higher order resources such as cognition are required to complete this process of sensory reweighting [36]. Similar to elderly people, patients with Parkinson’s disease and patients who suffered from stroke, symptomatic PVD patients with vestibular dysfunction may have a higher cognitive load [36]. A systematic review on the effect of bilateral vestibulopathy on attention supports these findings [37]. Adding a cognitive dual-task to a postural task results in poorer performance compared to a single postural task in patients with bilateral vestibulopathy [37]. However, whether patients with an uncompensated vestibular deficit, and more specifically a positive VOR phase, indeed use alternative and less efficient neural networks, needs to be established with appropriate methods, i.e. neural imaging such as functional MRI.

Next, by applying logistic regression to label patients as either compensated or uncompensated, the AVeCI was created which allows to stage the degree of compensation, rather than just classification. This tool is in particular of great use to evaluate VR. It is based on the Romberg with Jendrassik maneuver, standing on foam, tandem stance and one-leg stance, all with eyes closed condition. The seconds summed for each of these tasks, with a maximum of 30 s per item, added with the age of the patient, yield a simple number that is either positive or negative. The more positive, the better compensated, the more negative the worse a given patient is compensated. This AVeCI is much more than a group description. It allows the clinician or physical therapist to assess the degree of compensation and the evolution of the VR. Its clinical usefulness and relationship with overall well-being and quality of life however, needs to be investigated in future research.

This study has some limitations. Although the present sample is quite heterogeneous with respect to etiology (Table 2), in each group the majority of the patients had a diagnosis of vestibular schwannoma. Ninety percent of the vestibular schwannoma patients were assessed preoperatively. Patients with vestibular schwannomas become symptomatic once the vestibular loss becomes excessive [35]. These patients usually compensate centrally during the progressive emergence of the schwannomas on the vestibular nerve branch, but also because the severity of the vestibular loss seems to be less severe than in case of acute PVD [11]. In these vestibular schwannoma patients, the tumor size determines the degree of symptoms both regarding vestibular function and regarding balance tends [35]. However, this important information was missing and could therefore not be corrected for.

In conclusion, the AVeCI can be particularly useful in primary care. It allows physicians to easily screen a patient for referral and for physiotherapists to swiftly identify uncompensated PVD patients as they might benefit from VR [12]. They do not have specific laboratory equipment for assessing vestibular function at their disposal. But the use of the AVeCI compensates for the lack of these specific laboratory equipment and provides a tool to assess the vestibular compensation based on readily available measures.

## References

[1] Allum JH, Honegger F (2013). Relation between head impulse tests, rotating chair tests, and stance and gait posturography after an acute unilateral peripheral vestibular deficit. Otol Neurotol.

[2] Allum JH, Honegger F (2016). Recovery times of stance and gait balance control after an acute unilateral peripheral vestibular deficit. J Vestib Res.

[3] Kammerlind AS, Ledin TE, Odkvist LM, Skargren EI (2006). Influence of asymmetry of vestibular caloric response and age on balance and perceived symptoms after acute unilateral vestibular loss. Clin Rehabil.

[4] Lacour M, Bernard-Demanze L (2015). interaction between vestibular compensation mechanisms and vestibular rehabilitation therapy: 10 recommendations for optimal functional recovery. Front Neurol.

[5] Regrain E, Regnault P, Kirtley C, Shamshirband S, Chays A, Boyer FC, Taiar R (2016). Impact of multi-task on symptomatic patient affected by chronical vestibular disorders. Acta Bioeng Biomech.

[6] Whitney SL, Alghwiri AA, Alghadir A (2016). An overview of vestibular rehabilitation. Handb Clin Neurol.

[7] Van Der Stappen A, Wuyts FL, Van De Heyning PH (2000). Computerized electronystagmography: normative data revisited. Acta Otolaryngol.

[8] Rosengren SM, Welgampola MS, Colebatch JG (2010). Vestibular evoked myogenic potentials: past, present and future. Clin Neurophysiol.

[9] Allum JHJ, Scheltinga A, Honegger F (2017). The effect of peripheral vestibular recovery on improvements in vestibulo-ocular reflexes and balance control after acute unilateral peripheral vestibular loss. Otol Neurotol.

[10] MacDougall HG, Weber KP, McGarvie LA, Halmagyi GM, Curthoys IS (2009). The video head impulse test: diagnostic accuracy in peripheral vestibulopathy. Neurology.

[11] Halmagyi GM, Weber KP, Curthoys IS (2010). Vestibular function after acute vestibular neuritis. Restor Neurol Neurosci.

[12] Cerchiai N, Navari E, Sellari-Franceschini S, Re C, Casani AP (2018). Predicting the outcome after acute unilateral vestibulopathy: analysis of vestibulo-ocular reflex gain and catch-up saccades. Otolaryngol Head Neck Surg.

[13] Okinaka Y, Sekitani T, Okazaki H, Miura M, Tahara T (1993). Progress of caloric response of vestibular neuronitis. Acta Otolaryngol Suppl.

[14] Hall CD, Herdman SJ, Whitney SL, Cass SP, Clendaniel RA, Fife TD, Furman JM, Getchius TS, Goebel JA, Shepard NT, Woodhouse SN (2016). Vestibular rehabilitation for peripheral vestibular hypofunction: an evidence-based clinical practice guideline: from the american physical therapy association neurology section. J Neurol Phys Ther JNPT.

[15] Evans MK, Krebs DE (1999). Posturography does not test vestibulospinal function. Otolaryngol Head Neck Surg.

[16] Horak FB (2006). Postural orientation and equilibrium: what do we need to know about neural control of balance to prevent falls?. Age Ageing.

[17] Jacobson GP, McCaslin DL, Piker EG, Gruenwald J, Grantham S, Tegel L (2011). Insensitivity of the “Romberg test of standing balance on firm and compliant support surfaces” to the results of caloric and VEMP tests. Ear Hear.

[18] Cohen HS, Kimball KT (2008). Usefulness of some current balance tests for identifying individuals with disequilibrium due to vestibular impairments. J Vestib Res.

[19] Wuyts FL, De Bodt MS, Molenberghs G, Remacle M, Heylen L, Millet B, Van Lierde K, Raes J, Van de Heyning PH (2000). The dysphonia severity index: an objective measure of vocal quality based on a multiparameter approach. J Speech Lang Hear Res.

[20] Maes L, Vinck BM, Wuyts F, D'Haenens W, Bockstael A, Keppler H, Philips B, Swinnen F, Dhooge I (2011). Clinical usefulness of the rotatory, caloric, and vestibular evoked myogenic potential test in unilateral peripheral vestibular pathologies. Int J Audiol.

[21] Maes L, Dhooge I, D'Haenens W, Bockstael A, Keppler H, Philips B, Swinnen F, Vinck BM (2010). The effect of age on the sinusoidal harmonic acceleration test, pseudorandom rotation test, velocity step test, caloric test, and vestibular-evoked myogenic potential test. Ear Hear.

[22] Wall C, Black FO, Hunt AE (1984). Effects of age, sex and stimulus parameters upon vestibulo-ocular responses to sinusoidal rotation. Acta Otolaryngol.

[23] Whitney SL, Wrisley DM, Brown KE, Furman JM (2004). Is perception of handicap related to functional performance in persons with vestibular dysfunction?. Otol Neurotol.

[24] Jacobson GP, Newman CW (1990). The development of the Dizziness Handicap Inventory. Arch Otolaryngol Head Neck Surg.

[25] Fong E, Li C, Aslakson R, Agrawal Y (2015). Systematic review of patient-reported outcome measures in clinical vestibular research. Arch Phys Med Rehabil.

[26] Vereeck L, Truijen S, Wuyts F, Van de Heyning PH (2006). Test-retest reliability of the Dutch version of the Dizziness Handicap Inventory. B ENT.

[27] Vereeck L, Truijen S, Wuyts FL, Van De Heyning PH (2007). Internal consistency and factor analysis of the Dutch version of the Dizziness Handicap Inventory. Acta Otolaryngol.

[28] Vereeck L, Truijen S, Wuyts FL, Van de Heyning PH (2007). The Dizziness Handicap Inventory and its relationship with functional balance performance. Otol Neurotol.

[29] Vereeck L, Wuyts F, Truijen S, Van de Heyning P (2008). Clinical assessment of balance: normative data, and gender and age effects. Int J Audiol.

[30] Podsiadlo D, Richardson S (1991). The Timed Up and Go: a test of basic functional mobility for frail elderly persons. J Am Geriatr Soc.

[31] Whitney SL, Marchetti GF, Schade A, Wrisley DM (2004). The sensitivity and specificity of the Timed “Up and Go” and the Dynamic Gait Index for self-reported falls in persons with vestibular disorders. J Vestib Res.

[32] Shumway-Cook A, Woollacott M, Shumway-Cook A, Woollacott MH (1995). Assessment and treatment of the patient with mobility disorders. Motor control: theory and practical applications.

[33] Whitney SL, Hudak MT, Marchetti GF (2000). The dynamic gait index relates to self-reported fall history in individuals with vestibular dysfunction. J Vestib Res.

[34] Hinkle DE, Wiersma W (2003). Applied statistics for the behavioral sciences.

[35] Gouveris H, Helling K, Victor A, Mann W (2007). Comparison of electronystagmography results with dynamic posturography findings in patients with vestibular schwannoma. Acta Otolaryngol.

[36] Micarelli A, Viziano A, Della-Morte D, Augimeri I, Alessandrini M (2018). Degree of functional impairment associated with vestibular hypofunction among older adults with cognitive decline. Otol Neurotol.

[37] Dobbels B, Peetermans O, Boon B, Mertens G, Van de Heyning P, Van Rompaey V (2019). Impact of bilateral vestibulopathy on spatial and nonspatial cognition: a systematic review. Ear Hear.

